# A Bacteriophage Cocktail Reduces Five Relevant *Salmonella* Serotypes at Low Multiplicities of Infection and Low Temperatures

**DOI:** 10.3390/microorganisms11092298

**Published:** 2023-09-12

**Authors:** Tamar Gvaladze, Hansjörg Lehnherr, Julia Große-Kleimann, Stefan Hertwig

**Affiliations:** 1 Department Biological Safety, German Federal Institute for Risk Assessment, 10589 Berlin, Germany; tamar.gvaladze@bfr.bund.de; 2 Phage Technology Center GmbH, 59199 Boenen, Germany; h.lehnherr@ptc-phage.com; 3 Department for Biometry, Epidemiology and Information Processing, University of Veterinary Medicine Hannover, Foundation, 30559 Hannover, Germany; julia.grosse-kleimann@tiho-hannover.de

**Keywords:** *Salmonella*, foodborne zoonosis, phage, biocontrol, application

## Abstract

*Salmonella* are important pathogenic bacteria and, following *Campylobacter*, they are the second most common cause of bacterial foodborne infections worldwide. To reduce the presence of bacteria along the food chain, the application of bacteriophages (phages) may be a promising tool. In this study, the lytic properties of six phages against five relevant *Salmonella* serotypes (*S.* Enteritidis, *S*. Typhimurium, *S*. Infantis, *S*. Paratyphi B and *S*. Indiana) were analyzed. Three phages were able to lyse all five serotypes. We determined the lytic potential of each phage on indicator strains in vitro at room temperature (RT) and at 37 °C using low multiplicities of infection (MOIs). Most phages reduced their host more efficiently at RT than at 37 °C, even at the lowest MOI of 0.001. Following this, the lytic activity of a cocktail comprising five phages (MOI = 0.1) was examined with each of the five serotypes and a mix of them at RT, 15, 12, 10, 8 and 6 °C. All cultures of single serotypes as well as the mixture of strains were significantly reduced at temperatures as low as 8 °C. For single serotypes, reductions of up to 5 log_10_ units and up to 2.3 log_10_ units were determined after 6 h (RT) and 40 h (8 °C), respectively. The mixture of strains was reduced by 1.7 log_10_ units at 8 °C. The data clearly suggest that these phages are suitable candidates for biocontrol of various *Salmonella* serotypes under food manufacturing conditions.

## 1. Introduction

*Salmonella* is a genus of Gram-negative, rod-shaped bacteria belonging to the family of *Enterobacteriaceae* [1]. The genus contains two different species, *S. enterica* and *S. bongori*. *Salmonella* is one of the major causes of bacterial gastroenteritis worldwide [2,3,4], and salmonellosis is the third leading reason of human deaths among foodborne diseases [5]. Non-typhoid *Salmonella* cause 150 million enteric infections leading to 60,000 deaths worldwide each year [6]. Poultry, especially chicken, is the most common reservoir for *Salmonella* [5,7,8,9,10,11]. Thus, *Salmonella* is of major importance for public and animal health [10,12]. In the European Union (EU), the general trend for salmonellosis stayed constant during the last five years. *Salmonella* remained the second most commonly reported foodborne cause of gastroenteritis. In 2021, the number of human cases was reported to be 60,050 [7]. The species *S. enterica* and *S*. *bongori* comprise about 2600 serotypes. *S*. *enterica* is divided into six subspecies, the largest of which is *S*. *enterica* subsp. *Enterica* with over 1500 serotypes [13,14]. This subspecies is most important for human infections, particularly its serotypes Enteritidis and Typhimurium [14,15,16,17]. In the EU, these two serotypes are responsible for over 70% of human cases [7] and they are mainly associated with poultry, especially *S*. Enteritidis [7,18,19,20]. However, bacterial contamination on broiler farms is increasingly related to the *S*. Infantis serotype [21,22,23]. Other relevant serotypes associated with chicken meat in Germany and Vietnam are *S*. Paratyphi B and *S*. Indiana, respectively [24,25]. Among the foodborne outbreaks reported in the EU in 2021, *Salmonella* represented the largest percentage, at 19%, and *S*. Enteritidis caused the majority (80%) of outbreaks [7].

Chicken meat is the most frequently consumed meat product worldwide and many antibiotics are used during its production in developed countries [26]. The increasing consumption of chicken meat raises the risk of exposure to *Salmonella* from contaminated food [10]. Hence, the biocontrol of *Salmonella* is particularly important in chicken, as well as in other live animals and derived food products.

There are many antimicrobial methods available, such as chemical and physical treatments. However, they have the disadvantage of the possibility of changing the organoleptic characteristics of food. Additionally, the usage of these treatments kills not only pathogens, but also bacteria beneficial for humans [27]. One of the most common and effective antimicrobial methods is the use of antibiotics. Unfortunately, nowadays, antibiotic resistance is a serious problem for the whole world [28]. In the EU in 2021, high levels of human isolated *Salmonella* strains resistant to three or more antimicrobials were reported [29]. Therefore, there is an urgent need to develop an alternative antimicrobial approach.

One natural tool to control or reduce bacteria could be the application of bacteriophages (phages) [30]. Phages are found in large numbers in all environments such as water, soil, food, and in the intestine of humans and animals [31]. In total, there are about ten times more phages than bacteria in the biosphere [32] and they kill up to 40% of all bacteria in the oceans daily [33]. Thus, phages play an important role in the microbial balance in nature [34]. Phages are natural agents against bacteria, which they infect and lyse very specifically, as they are mostly able to kill only single or closely related species [35]. Depending on their life cycle, phages can be virulent or temperate. Virulent phages always undergo a lytic cycle that ends with the lysis of the bacterial cell, whereas temperate phages, in addition to a lytic cycle, have a second developmental pathway called a lysogenic cycle. At the lysogenic stage, temperate phages integrate their genome into the bacterial chromosome as a prophage and are replicated passively, together with the bacterial chromosome [36]. Therefore, generally, only virulent phages are suitable for an antimicrobial approach to combat bacterial contamination [27,37]. Such phages can be used specifically against certain pathogenic bacteria [38]. In this study, our focus was on *Salmonella*, as these bacteria have a relevant reservoir in poultry [39]. Phages could be applied during all phases of poultry production “from farm to fork”. Post-harvest application of phages could be employed for food biocontrol or disinfection of food contact or nonfood contact surfaces [40].

The temperature and duration of phage exposure, phage dose and the use of a single phage or a mix of phages are important parameters and play key roles for a successful application in a food production setting [1]. Therefore, before being able to use and successfully apply phages, it is necessary to characterize them for their suitability. Many publications report on phage reduction experiments in liquid culture, before applying the phages to food samples [41,42,43,44]. However, most studies were performed at 37 °C using single *Salmonella* serotypes, which were infected by phages at high multiplicities of infection (MOIs).

In this study, we evaluated important parameters for the practical application of six *Salmonella* phages. In addition to host range and efficiency of plating experiments, we also determined the reduction efficiency of single phages and a phage cocktail in liquid culture. The main focus was to study the reduction capacity of these phages at different temperatures on five important *Salmonella* serotypes. We chose several temperatures to determine the step of the food production chain at which phage application would be most suitable. Thus far, most studies reported on the use of *Salmonella* phages against *S*. Enteritidis and *S*. Typhimurium [45], but, as mentioned above, there are other serotypes with relevance, especially in connection with chicken meat. Therefore, we carried out our investigations with the five serotypes, *S*. Enteritidis, *S*. Typhimurium, *S*. Infantis, *S*. Paratyphi B and *S*. Indiana.

## 2. Materials and Methods

### 2.1. Bacterial Strains and Growth Conditions

For the propagation of the phages, six indicator strains were used (Table 1). These strains were isolated between 2009 and 2011 from various animal facilities in Germany and Italy. The determination of the host range was performed with 20 additional *Salmonella* strains, four of them each belonging to the serotypes *S*. Enteritidis, *S*. Typhimurium, *S*. Infantis, *S*. Paratyphi B and *S*. Indiana (Table 2). The selection was based on the year of isolation and matrix. These strains were obtained from the strain collection of the National Reference Laboratory for *Salmonella* at the German Federal Institute for Risk Assessment, (BfR) Berlin, Germany. They originated from chicken meat and skin samples, collected in slaughter houses and retail between 2019 and 2020. All *Salmonella* strains were serotyped using poly- and monovalent anti-O as well as anti-H sera (Sifin diagnostics GmbH, SIFIN, Berlin, Germany) according to the White–Kauffmann–Le Minor scheme [46]. *Salmonella* stock cultures were stored at −80 °C. *Salmonella* strains were cultivated on a 1.5% lysogeny broth (LB; Carl Roth GmbH, Karlsruhe, Germany) agar prepared according to the manufacturer’s instructions at 37 °C overnight. Thereafter, one colony was used for a subsequent culturing in LB broth at 37 °C overnight.

### 2.2. Origin of the Salmonella Phages

The six phages investigated are components of a commercially available product (FinkTec GmbH, Boenen, Germany), used to fight *Salmonella* in both meat (GRN 001038) [47] and vegetable (GRN 001070) processing [48]. The phages were isolated from environmental sources (duck pond, Hamm, Germany, and sewage treatment plant, Hamm, Germany) between 2009 and 2010.

### 2.3. Propagation and Enumeration of Phages

High-titer lysates of the phages were produced by infecting 200 mL cultures of the respective indicator strain (Table 1) at an optical density at a wavelength of 588 nanometers (OD_588_) of 0.2. Phages were at an MOI of 0.1 and incubated at 37 °C. Alternatively, 10 agar plates were prepared with a confluent lysis of the indicator strain using the conventional overlay method [49]. In this case, after incubation overnight at 37 °C, the soft agar (0.6%) was scraped off from plates, mixed with a 200 mL sodium magnesium buffer (SM, 50 mM Tris-HCL, a 100 mM sodium chloride, a 8 mM magnesium sulfate, pH 7.5) and stirred for up to four hours. Lysates were centrifuged at 11,000× *g* for 20 min and the supernatant was filtered through a 0.22 µm bottle top filter (Corning GmbH, Kaiserslautern, Germany). Subsequently, the enumeration of phages was conducted using the double agar overlay plaque assay [50].

### 2.4. Electron Microscopy of Bacteriophages

Electron microscopy of bacteriophages was conducted following the methodology described by Akhwale et al. (2019) [51].

### 2.5. Determination of the Host Range and Efficiency of Plating

The host range was determined using a spot dilution assay [52]. Briefly, 100 µL of overnight cultures of the respective *Salmonella* strains were mixed with 5 mL LB soft agar (0.6%) and poured on an LB agar plate. After solidifying, 10 µL of a serial dilution of the phage lysate were spotted onto the surface of the plate. The plates were incubated at 37 °C overnight.

The efficiency of plating (EOP) procedure was performed using the double agar overlay plaque assay [50]. After incubation overnight, the phage titers on the indicator strain and the tested strains were calculated and divided by each other.

### 2.6. Influence of the MOI on Single-Phage-Induced Lysis

An overnight culture of the indicator strain was transferred to fresh 20 mL LB broth and grown to an OD_588_ of 0.2, corresponding to approximately 5 × 10^7^ to 1 × 10^8^ CFU/mL. The culture was then divided into four equal portions, and three of them were infected with phages at an MOI of 0.1, 0.01, or 0.001. One tube was used as a control without added phages. After inoculation, the OD_588_ values of the cultures were measured every 30 min, until the difference between the OD_588_ values of the controls and the phage-treated cultures was the greatest. To determine the cell numbers, 100 µL of a serial dilution of the control and phage-treated culture were plated on LB agar plates, which were incubated overnight at 37 °C. The following day, colonies were counted and the difference between the controls and phage-treated cultures was calculated. The experiments were performed twice at 37 °C and at room temperature (RT, approx. 22 °C) with the six phages and their respective indicator strain or other strains that showed high EOP in the previous experiments (Table 3).

### 2.7. Influence of the Temperature on the Lysis by the Phage Cocktail

We selected five out of six phages for a cocktail based on the results of individual phage activity. As described above, for this experiment, the bacteria were grown to an OD_588_ of 0.2 (RT) or 0.1 (15, 12, 10, 8 and 6 °C). After reaching the respective OD_588_, the cultures were divided into two equal portions. One of them was inoculated with a phage cocktail, with each of the five phages at an MOI of 0.1. The other portion was used as a control without the addition of a 3phage cocktail. The OD_588_ values were measured every 30 min (RT) and every hour (lower temperatures) on the first day of the experiment, which was performed for 24 h until day 9, depending on the temperature and strain, which affect the bacterial growth. After reaching the biggest difference between the controls and the phage-treated cultures, cell counts were determined as described above. The temperature experiments were performed with each of the 10 *Salmonella* strains individually and with a mixed culture containing all 10 strains.

### 2.8. Determination of Phage Resistance

To examine possible resistance of the *Salmonella* strains, 20 colonies of each strain that survived infection by the phage cocktail at RT were isolated. Ten of the colonies were isolated after 6 or 7 h of phage infection. The remaining 10 colonies were isolated after infection by the phage cocktail overnight. Colonies were inoculated in an LB broth and incubated overnight at 37 °C. A total of 100 µL of the culture were mixed with a 5 mL LB soft agar (0.6%) and poured on LB agar. Thereafter, serial dilutions (10 µL) of each phage and of the cocktail were spotted. The plates were incubated overnight at 37 °C. The next day, the plates were analyzed for plaques.

## 3. Results

### 3.1. Morphology of the Six Salmonella Phages

**Figure 1:**
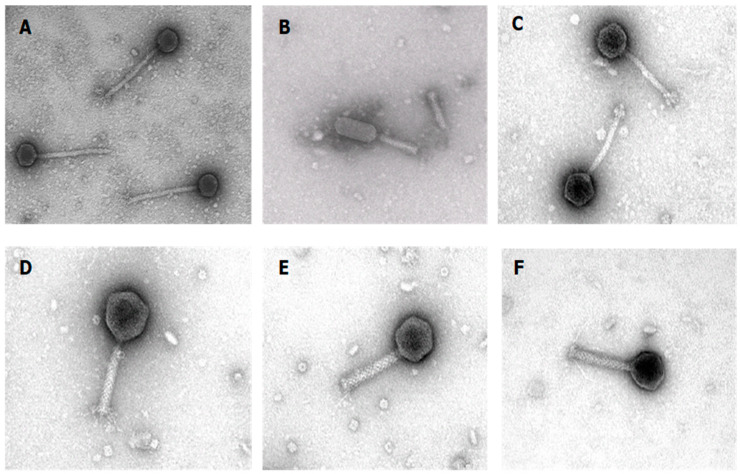
Electron micrographs of the six bacteriophages investigated in this study. (**A**) RMP9; (**B**) DIN2; (**C**) OBO18; (**D**) MP82; (**E**) TAT2F and (**F**) RMS3b. Electron micrographs were provided by Manfred Rohde from the Helmholtz Centre for Infection Research GmbH in Braunschweig, Germany.

Electron microscopic analyses revealed that four phages have a myoviridal morphology with a contractile tail. While the head of MP82, TAT2F and F-RMS3b is isodiametric, that of DIN2 is prolate. By contrast, the two phages RMP9 and OBO18 have a siphoviridal morphology with a long non-contractile tail (Figure 1).

### 3.2. Host Range

The host range of the six phages was examined on a total of 20 *Salmonella* strains belonging to five different serotypes, *S*. Enteritidis, *S*. Typhimurium, *S.* Indiana, *S*. Infantis and *S*. Paratyphi B (Table 2). Lysates of the phages exhibiting high titers (10^8^–10^9^ PFU/mL) were subjected to a tenfold serial dilution (10^−1^ to 10^−6^) and spotted onto soft agar containing the respective serotype. The phages revealed different host ranges. Table 4 shows that three phages (RMS3b, MP82 and TAT2F) lysed all five different *Salmonella* serotypes. Moreover, phage MP82 was able to lyse most strains (18/20). Phage DIN2 lysed all serotypes besides *S*. Indiana. By contrast, phage RMP9 and OBO18 showed lytic activity only on *S*. Typhimurium and *S*. Enteritidis/*S*. Indiana, respectively (Table 4).

Thus, four *Salmonella* phages were found to lyse at least 65% of the selected *Salmonella* strains. Moreover, a combination of phages MP82 and TAT2F was able to lyse all strains. It is noteworthy that *S*. Enteritidis was identified as the most susceptible serotype, while *S*. Infantis was shown to be insensitive to the largest number of phages. Nevertheless, all strains of *S*. Infantis were lysed by MP82 and DIN2 and every single strain was lysed by at least two phages.

### 3.3. Efficiency of Plating

To determine the efficiency of plating (EOP) of the phages, we chose ten *Salmonella* strains belonging to the five different serotypes. Each serotype was represented by two strains exhibiting different susceptibilities. As shown in Table 5, TAT2F, DIN2 and RMP9 revealed the highest EOP values between 0.1 and 10 on almost all *Salmonella* strains that were lysed by these phages. On the other hand, RMS3B and MP82 achieved maximum values between 0.1 and 1, whereas the EOP of OBO18 was even lower.

Even though the phages MP82 and TAT2F have a very similar host range, their EOPs differed. While MP82, e.g., reached EOPs between 0.000001 and 0.0001 on *S*. Indiana, TAT2F showed much higher EOPs between 0.1 and 1. OBO18 lysed only both strains of *S.* Enteritidis and one strain of *S.* Indiana with an EOP of up to 0.1. The EOP values of OBO18 on *S*. Enteritidis strains differed significantly. In contrast, RMS3b revealed similar EOP values on strains belonging to the same serotype. In conclusion, each strain was lysed by at least one phage with an EOP between 0.1 and 1 (Table 5).

### 3.4. Five Phages Were Highly Active at Room Temperature and at Low MOIs

To assess their potential application, phage-induced lysis of the *Salmonella* strains was analyzed in detail. We studied the lytic activity of the phages in liquid culture using three different MOIs (0.1, 0.01 and 0.001) and two temperatures (RT and 37 °C). For these experiments, either the indicator strains applied for the propagation of the phages (see M + M) or strains that showed an even higher EOP in the above experiments were used (Table 5).

**Figure 2:**
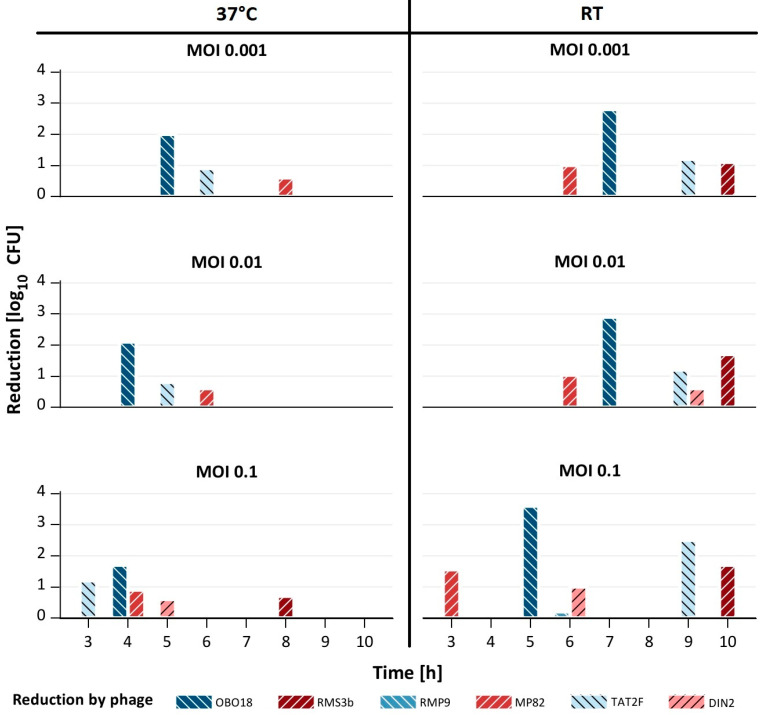
Reduction in selected Salmonella strains by single phages. The figure displays the reduction by single phages at 37 °C and room temperature (RT) using different multiplicities of infection (MOIs). The strains are not shown here (see Table 3).

Figure 2 shows that except for RMP9, all phages were able to significantly (0.6–3.6 log_10_ units) reduce the tested strains at both temperatures using a MOI of 0.1, even though the bacterial reduction was delayed at RT, compared to 37 °C. For example, with TAT2F, the time needed for bacterial lysis was delayed by 3 to 6 h. However, reduction by the five phages DIN2, MP82, OBO18, RMS3b and TAT2F was much stronger at RT than at 37 °C (Figure 2).

At an MOI of 0.01, similar reductions were obtained with the five phages at RT, whereas only three of them efficiently lysed their respective hosts at 37 °C. Using an MOI of 0.001, three and four phages were still able to reduce the *Salmonella* strains significantly (up to 2.8 log_10_ units) at 37 °C and RT, respectively. In conclusion, five phages were able to lyse their hosts at both temperatures, when an MOI of 0.1 was applied, while at the lower MOIs, much better results were obtained at RT.

### 3.5. A Cocktail Comprising Five Phages Significantly Reduced Five Salmonella Serotypes and a Mixture of Them, Even at Low Temperatures

Based on the lytic activity of five phages in liquid culture, they were studied as part of a cocktail. The experiments were again performed in liquid at low temperatures (RT, 15, 12, 10, 8 and 6 °C) using an MOI of 0.1. At RT, we treated the above-mentioned 10 *Salmonella* strains individually as well as a mixture of them with the phage cocktail.

**Figure 3:**
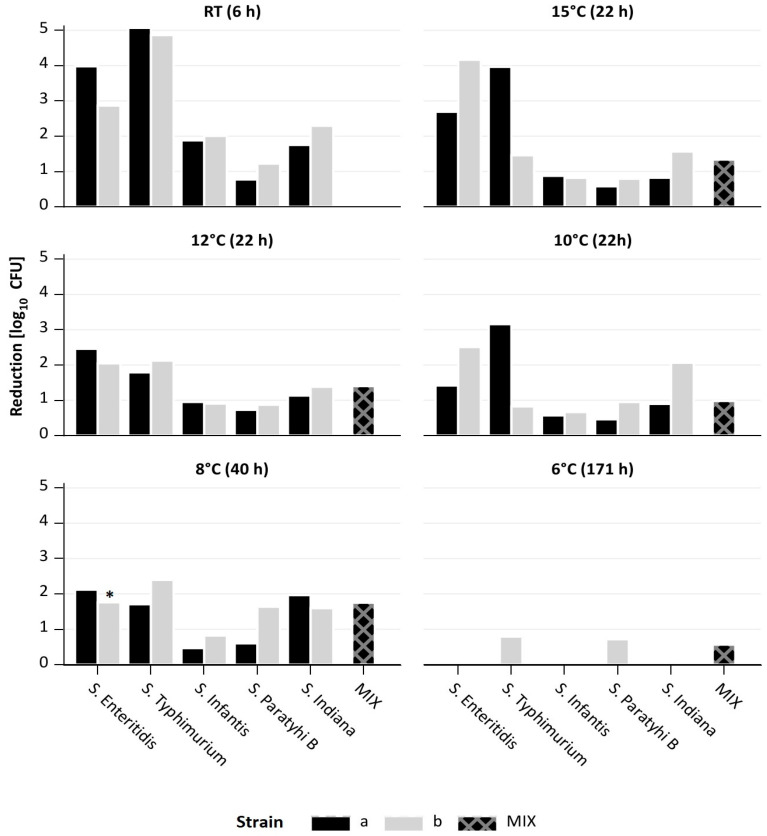
Reduction in different *Salmonella* serotypes, alone or in a mixture, by a 5-phage cocktail at different temperatures using multiplicities of infection (MOI) of 0.1. *—135 h after treatment.

Figure 3 shows that at RT, significant reductions between 0.8 and 5.1 log_10_ units were achieved with all tested *Salmonella* strains after 6 h of phage treatment. The strongest reductions were determined with *S*. Enteritidis and *S*. Typhimurium, the lowest with *S*. Paratyphi B. After 24 h, very similar results were obtained (Table S1). To determine the resistance development of surviving bacteria after 6 or 24 h of treatment with the phage cocktail, the phage sensitivity of 10 colonies of each treated culture was determined. Resistance varied depending on the strain or phage, but resistance to all phages of the cocktail was not observed. There was always at least one phage to which the bacteria showed sensitivity (Table S1). At lower temperatures, longer incubation times were required, since the growth of the bacteria was slower. Nevertheless, at 15, 12, and 10 °C, significant reductions between 0.5 and 4.2 log_10_ units were achieved after 22 h of phage treatment. The strongest reductions were again determined with *S*. Enteritidis and *S*. Typhimurium. Moreover, a mixture of the 10 *Salmonella* strains was also reduced by 1 to 1.4 log_10_ units after phage treatment. The threshold temperature at which lysis of the strains was detected was 8 °C. Here, an incubation of at least 40 h was required to reduce the single strains by 0.5 to 2.4 log_10_ units and the mixture by 1.4 log_10_ units. At 6 °C, growth inhibition was observed with most strains. We determined the reduction only when the OD values between control and treated samples differed significantly. However, besides *S*. Typhimurium and *S*. Paratyphi B, the mix culture was reduced by almost 1 log_10_ units at this very low temperature after incubation for 171 h (Figure 3).

## 4. Discussion

In this study, we characterized six *Salmonella* phages in terms of their potential to reduce five different *Salmonella* serotypes (*S.* Enteritidis, *S*. Typhimurium, *S*. Infantis, *S*. Paratyphi B and *S*. Indiana) which are currently of epidemiological importance. Most studies on *Salmonella* phages published thus far focused on *S.* Enteritidis and *S*. Typhimurium, while other serotypes have only rarely been investigated. To our knowledge, there are still only three publications on the reduction in *S*. Infantis by phages and two other reports, where *S*. Paratyphi B was examined as part of a mixture of strains [38,53,54,55,56]. Moreover, phages infecting *S*. Indiana have only been analyzed regarding their host specificity [44,57]. Four of our phages were able to lyse all five serotypes, most of them with high efficiency. Thus, these four phages and phage OBO18 were used as a cocktail for some of our reduction experiments. We, however, determined first the reduction in indicator strains by each individual phage quantitatively using MOIs between 0.001 and 0.1 at two temperatures (RT and 37 °C). It is noteworthy that reductions at RT were at least as strong as at 37 °C, regardless of the applied MOI, even though the time needed for reduction was longer at RT. Most other studies published thus far were performed at 37 °C or used high MOIs of single phages. Hungaro et al. (2013), for example, investigated five phages at 25 and 37 °C and found a significant growth inhibition of the *S*. Enteritidis strain at both temperatures only with an MOI of 10, whereas no reduction was observed with MOIs of 0.00001 and 0.01 [41]. A high MOI (10^4^/10^5^) was required at 4 °C compared to 37 °C (MOI 0.0001 to 10) to achieve a reduction between 1.4 and 3 log_10_ units of *Salmonella* (*S*. Typhimurium strains) in LB [44]. Similar results were reported by Yamaki et al. (2022) and Wang et al. (2017) who achieved a reduction in the *S*. Typhimurium strain at 4, 25 and 37 °C using an MOI of 10^4^ and 10^6^, respectively, while in the latter study, an MOI of one was not sufficient to reduce the bacteria at 4 and 25 °C [58,59]. MOIs of 100 and higher were also applied by other authors who demonstrated a killing effect by single *Salmonella* phages [60,61,62,63]. On the other hand, in two studies, MOIs between 0.1 and 10 and between 0.01 and 100 resulted in similar reductions at 37 °C [64,65]. Nevertheless, to the best of our knowledge, this is the first report describing phages lysing five relevant *Salmonella* serotypes at different temperatures using low MOIs, which are suitable for applications under food chain conditions.

Based on these promising data, we analyzed the reduction in single *Salmonella* strains as well as a mixture of strains by a phage cocktail containing five of the phages at different temperatures using an MOI of 0.1. Here, we could clearly demonstrate that a cocktail was able to lyse both single strains and the mixture of *Salmonella* strains efficiently after incubation for one or two days, even at a temperature as low as 8 °C. Particularly *S*. Enteritidis and *S*. Typhimurium were strongly reduced. These two serotypes have previously been used individually for studies with phage cocktails, even though most of them were again exclusively carried out at 37 °C and by applying high MOIs. Kim et al. (2020) examined the lytic activity of four phages on a cocktail of three *S.* Enteritidis strains using a MOI of 10^4^ and determined a reduction of approximately 3 log_10_ units [62]. Petsong et al. (2019) reported a reduction of 4 log_10_ units of *S*. Enteritidis and *S*. Typhimurium strain by three phages that were applied at an MOI of 100 [66]. Similarly, growth inhibitions of *S*. Enteritidis and/or *S*. Typhimurium strain were determined in studies of Islam et al. (2019) and Esmael et al. (2021) who used three phages at MOIs between 0.1 and 100 and two phages at MOIs between 0.01 and 10, respectively, at 37 °C [42,67]. By contrast, there are only few publications on the use of a phage cocktail at low temperatures. In one study, reductions in *S*. Enteritidis and *S*. Typhimurium strains of up to 4.9 log_10_ units at 25 °C and up to 2.6 log_10_ units at 8 °C by five phages (MOI 10^4^) have been reported [43]. The commercial preparation “SalmoFreshTM” (Intralytix Inc., Columbia, MD, USA) comprising six phages was shown to reduce single cultures of *S*. Enteritidis *S*. Typhimurium and *S*. Heidelberg at 4 °C by 2.7 log_10_ units, when MOIs of 10^4^ and 10^5^ were applied. However, a mixture of five serotypes as in this study has not been examined before. Duc et al. (2020) and Wang et al. (2017) used a single phage at 4 °C with MOIs of 10^4^ and 10^5^ for reduction experiments with two and four *Salmonella* serotypes achieving reductions of up to 1.3 and 2.3 log_10_ units, respectively [59,68]. Finally, growth inhibition was reported for different combinations of *S*. Enteritidis, *S.* Paratyphi B and *S*. Typhimurium infected with three phages at 37 °C using an MOI of one [55].

In conclusion, our study showed that a phage cocktail containing five highly efficient phages was able to lyse a mixture of five *Salmonella* serotypes at a rather low MOI of 0.1 and at low temperatures. The highest reduction rates were found for *S*. Enteritidis and *S*. Typhimurium, the isolates which cause most foodborne infections. This makes the phage cocktail even more attractive for the biocontrol of *Salmonella*. Thus, this cocktail may be suitable for applications under conditions that are found in a slaughterhouse or during food processing, e.g., by spraying the cocktail on chicken carcasses or immersion.

## Figures and Tables

**Table 1:** Indicator strains for the propagation of the six *Salmonella* phages.

	Indicator Strain			
Phage	Serotype	Isolation Year	Origin	Country of Origin
OBO18	*S.* Enteritidis 20	2009	Chicken	Germany
RMS3b	*S.* Newport 115	2010	Turkey	Germany
RMP9	*S.* Typhimurium 9	2009	Chicken	Germany
MP82	*S.* Virchow 2	2009	Chicken	Germany
TAT2F	*S.* Derby 152	2011	Pig	Italy
DIN2	*S.* Java 14	2009	Chicken	Germany

**Table 2:** Origin and serotype of twenty *Salmonella* strains used for host range determination.

Serotype/Designation	Isolation Year	Strain N	Matrix
*S*. Enteritidis/a	2019	SA00115	Frozen raw chicken meat
*S*. Enteritidis/b	2020	SA02231	Poultry meat
*S*. Enteritidis/c	2020	SA00763	Frozen raw chicken meat
*S*. Enteritidis/d	2019	SA03612	Frozen raw chicken meat
*S*. Typhimurium/a	2020	SA01020	Chicken meat
*S*. Typhimurium/b	2020	SA02878	Frozen poultry meat
*S.* Typhimurium/c	2020	SA01009	Chicken meat
*S*. Typhimurium/d	2019	SA03116	Frozen chicken meat
*S*. Infantis/a	2020	SA02265	Frozen chicken meat
*S*. Infantis/b	2020	SA02511	Broilers; skin with fat
*S*. Infantis/c	2019	SA02535	Broilers; skin with fat
*S*. Infantis/d	2019	SA02825	Frozen chicken meat
*S*. Paratyphi B/a	2019	SA01081	Frozen chicken meat
*S*. Paratyphi B/b	2020	SA01326	Frozen chicken lower leg
*S*. Paratyphi B/c	2019	SA00866	Frozen chicken breast
*S*. Paratyphi B/d	2020	SA02251	Broilers; skin with fat
*S*. Indiana/a	2020	SA01985	Broilers; skin with fat
*S*. Indiana/b	2019	SA02184	Frozen raw chicken meat
*S*. Indiana/c	2020	SA02067	Frozen raw chicken meat
*S*. Indiana/d	2019	SA02269	Broilers; skin with fat

**Table 3:** Strains and phages used for initial reduction tests.

Phage	Strain
OBO18	* *S*. Enteritidis 20
RMS3b	* *S*. Newport 115
RMP9	* *S*. Typhimurium 9
MP82	* *S*. Virchow 2
TAT2F	** *S*. Typhimurium a
DIN2	** *S*. Infantis a

* Indicator strain. ** Strain with highest EOP.

**Table 4:** Host range of the six phages.

		Phage					
Serotype	*Salmonella* Strain	OBO18	RMS3b	RMP9	MP82	TAT2F	DIN2
*S.* Enteritidis	a	+	+++		+++	+++	+++
	b	++	+++		+	+++	+
	c	+	+		+++		+++
	d	+	+++		++	+++	+
*S.* Typhimurium	a		++	+	+++	+++	
	b		+++	+++	+++	+++	+
	c		+++	++	+++	+++	
	d		+++	++	+++	+++	
*S.* Infantis	a		+		+++		+++
	b				+++	+	+++
	c				+++		+++
	d				+++		+++
*S.* Paratyphi B	a				+	+++	+++
	b		+		+	+++	+++
	c				+	++	+++
	d		+			+++	+++
*S.* Indiana	a	+	+++		+	+++	
	b		+++		+	+++	
	c	+	+++			+++	
	d	+	+++		+	+++	
Bacteria Total (*n* = 20)		7/20	15/20	4/20	18/20	16/20	13/20

+++ High EOP (single plaques obtained with 10^−5^ and 10^−6^ dilutions). ++ Medium EOP (single plaques obtained with 10^−3^ and 10^−4^ dilutions). + Low EOP (single plaques obtained with 10^−1^ and 10^−2^ dilutions, mostly turbid plaques).

**Table 5:** Efficiency of plating of the six phages.

		Phage					
Serotype	*Salmonella* Strain	OBO18	RMS3b	RMP9	MP82	TAT2F	DIN2
*S.* Enteritidis	a						
	b						
*S.* Typhimurium	a						
	b						
*S.* Infantis	a						
	b						
*S.* Paratyphi B	a						
	b						
*S.* Indiana	a						
	b						

The table displays the Efficiency of Plating (EOP) of phages. The colors in the table represent the following: 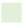 0.000001 ≤ EOP < 0.0001. 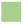 0.0001 ≤ EOP < 0.01. 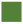 0.01 ≤ EOP < 0.1. 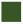 0.1 ≤ EOP < 1. 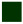 1 ≤ EOP < 10”.

## Data Availability

All data generated within this study are provided in the manuscript.

## Supplementary Materials

The following supporting information can be downloaded at: https://www.mdpi.com/article/10.3390/microorganisms11092298/s1, Table S1: Phage Resistance.
