# Supplementary material for: A Bacteriophage Cocktail Reduces Five Relevant Salmonella Serotypes at Low Multiplicities of Infection and Low Temperatures
Source: Microorganisms. 2023 Sep 12;11(9):2298. doi: 10.3390/microorganisms11092298 (PMC10535997; doi:10.3390/microorganisms11092298)
Supplement: Supplementary file 1 [file microorganisms-11-02298-s001.zip › Supplementary Files.pdf]

Table S1. Phage Resistance.

Time after phage treatment	Serotype	*Salmonella* Strain	Phage					
			OBO18	RMS3b	MP82	TAT2F	DIN2	Cocktail
6 h	*S*. Enteritidis	a	/	20 %	0	30 %	0	0
24 h	*S*. Enteritidis	a	/	100 %	80 %	100 %	60 %	80
6 h	*S*. Enteritidis	b	30 %	30 %	20 %	50 %	30 %	30 %
24 h	*S*. Enteritidis	b	20 %	60 %	60 %	60 %	100 %	20 %
6 h	*S*. Typhimurium	a	/	100 %	20 %	90 %	/	70 %
24 h	*S*. Typhimurium	a	/	100 %	70 %	100 %	/	60 %
6 h	*S*. Typhimurium	b	/	100 %	50 %	100 %	70 %	50 %
24 h	*S*. Typhimurium	b	/	100 %	90 %	90 %	90 %	90 %
6 h	*S*. Infantis	a	/	0	90 %	/	90 %	0
24 h	*S*. Infantis	a	/	0	90 %	/	90 %	0
6 h	*S*. Infantis	b	/	/	80 %	0	80 %	0
24 h	*S*. Infantis	b	/	/	30 %	0	30 %	0
6 h	*S*. Paratyphi B	a	/	/	100 %	0	100 %	0
24 h	*S*. Paratyphi B	a	/	/	80 %	0	100 %	0
6 h	*S*. Paratyphi B	b	/	0	60 %	0	60 %	0
24 h	*S*. Paratyphi B	b	/	10 %	40 %	0	40 %	0
6 h	*S*. Indiana	a	100 %	100 %	/	100 %	/	100 %
24 h	*S*. Indiana	a	90 %	100 %	/	100 %	/	90 %
6 h	*S*. Indiana	b	/	40 %	40 %	/	40 %	40 %
24 h	*S*. Indiana	b	/	90 %	90 %	/	90 %	80 %

The Table summarizes the experiments assaying the resistance development of different Salmonella serotypes (S. Enteritidis, S. Typhimurium, S. Infantis, S. Paratyphi B, and S. Indiana) against an individual phage or a phage cocktail. The symbols in the figure represent the following: "/" indicates that a serotype was not tested due to the absence of lytic activity, "0" signifies no resistance development, and "%" indicates the percentage of resistant colonies observed, out of 10 colonies tested.
